# Severe medial tibial plateau fractures: feasibility and functional and radiographic results of an extended approach with medial femoral epicondyle osteotomy

**DOI:** 10.1186/s13018-026-07100-y

**Published:** 2026-08-08

**Authors:** Hannah Gablac, Christian Arras, Peter Behrendt, Hendrik Fahlbusch, Clemens Galavics, Michael Hoffmann, Karl-Heinz Frosch, Matthias Krause

**Affiliations:** 1 Department of Orthopaedics and Trauma Surgery, Musculoskeletal University Center Munich (MUM), Ludwig Maximilian University of Munich, Munich, Germany; 2 Department of Trauma and Orthopaedic Surgery, University Medical Center Hamburg-Eppendorf, Hamburg, Germany; 3 Department of Trauma and Orthopaedic Surgery, University Medical Center Schleswig-Holstein, Kiel, Germany; 4 Department of Trauma, Orthopaedic Surgery and Sports Traumatology, Asklepios Klinik St. Georg, Hamburg, Germany; 5 Department of Trauma Surgery, Orthopaedics and Sports Traumatology, BG Hospital Hamburg, Hamburg, Germany

**Keywords:** Tibia, Fractures, Extended medial approach, Epicondyle, Osteotomy

## Abstract

**Introduction:**

This study aimed to evaluate the clinical and radiological outcomes of tibial plateau fractures involving the medial plateau by comparing an extended medial approach with medial femoral epicondyle osteotomy (med ECO) to a conventional approach without epicondyle osteotomy (non-ECO).

**Methods:**

A retrospective cohort study was conducted at two level one trauma-centers. All fractures of the medial tibial plateau (OA/OTA Type B or C) treated with med ECO were classified according to the AO/OTA and 10-segment classification using pre-operative CT scans. A comparative cohort study was performed against non-ECO patients based on age, sex, body mass index (BMI), injury mechanism, and fracture patterns. The quality of fracture reduction was determined by post-operative CT scans. Functional outcomes and postoperative complications were assessed over a minimum follow-up of 2 years.

**Results:**

Twenty-nine patients (mean age: 50.2 *±* 10.2 years, mean follow-up 71.65 *±* 18.9 months) were analyzed. Both groups consisted of patients with severe tibial plateau fractures, knee dislocations, high-velocity trauma and polytrauma and concomitant injuries requiring an average hospital stay of 24.9 *±* 12.4 days. Radiological outcomes showed a medial fracture step > 2 mm in 10 cases (med ECO *n* = 5, non-ECO *n* = 5) and a gap > 5 mm in 8 cases (med ECO *n* = 3, non-ECO *n* = 5). The mean radiological Rasmussen score was 13.8 *±* 2.8 (med ECO 14.1 *±* 2.8 vs. non-ECO 13.4 *±* 3.0, *p* > 0.05). The mean Knee Injury and Osteoarthritis Outcome Score (KOOS) was 60.4 *±* 16.9 (med ECO 61.3 *±* 12.7 vs. non-ECO 59.5 + 16.2, *p* > 0.05) and the International Knee Documentation Committee score (IKDC score) was 56.6 *±* 16.9 (med ECO 58.8 *±* 19.0 vs. non-ECO 52.6 *±* 14.4, *p* > 0.05).

**Conclusion:**

Both med ECO and non-ECO demonstrated good radiological results. No significant differences were observed in clinical and radiological outcomes during follow-up. However, severe medial tibial plateau fractures are devastating knee injuries, being a challenge to the functional outcome of patients due to multiple concomitant injuries. Thus, medial epicondyle osteotomy should be reserved for specific fractures, considering the added morbidity and invasiveness associated with the procedure.

**Level of evidence:**

Retrospective Cohort Study, Level of Evidence = III.

## Introduction

Fractures of the tibial plateau are rare but often complex and can result in severe functional impairment of the affected knee if not managed appropriately [1]. The variety of fracture morphologies with complex involvement of both articular surfaces and the high incidence of associated soft tissue injuries such as compartment syndrome, chondral, ligamentous and meniscal lesions can present a significant surgical challenge, especially for fracture types 41-B3 and 41-C3 according to the AO/OTA (Arbeitsgemeinschaft für Osteosynthesefragen / Orthopaedic Trauma Association) [2–4].

Near-anatomic articular reconstruction is deemed essential to preserve knee function after complex tibial plateau fractures [5]. Recent studies have emphasized that better range of motion, less pain and a higher KOOS are correlated with preserving postoperative intra-articular joint irregularity of less than 2.5 mm [5–7]. It is evident that patient age, BMI, fracture type and the utilization of locking plates do not consistently predict the efficacy of reconstruction and consequently cannot be regarded as definitive prognostic factors. Instead, precise anatomical reconstruction of the articular surface, preservation of the leg axis, tibial slope and tibial width, as well as stable fixation, are essential. Furthermore, good management of the frequently associated soft tissue injury is critical to prevent postoperative instability or deformity and to minimise the risk of post-traumatic knee osteoarthritis [5–8]. Impaired visualization of the articular surface by standard surgical approaches is the main risk factor for the latter [8–10]. Although adjunctive techniques such as intraoperative 3D scanning or fracturoscopy may improve visualization, a fracture-specific surgical approach with articular access is still required to facilitate durable fracture fixation of anatomic articular reconstruction [8–11].

In Type B and C fractures involving the medial plateau, the posteromedial approach shows only a small exposure of the posterior articular surface of 14.0 + 7.3%, not even exposing the entire PMM fragments according to the 10-segment classification [12]. The feasibility and excellent postoperative reduction of the lateral segments in complex fractures and a good with with an overall low complication rate using the extended lateral approach with lateral epicondyle osteotomy has already been confirmed in recent reports [13, 14]. However, little is known about the clinical outcome in patients with complex fractures of the medial tibial plateau treated with medial epicondyle osteotomy (med ECO) [15]. The aim of this study was to clinically demonstrate the feasibility of the concept of extended medial access and improved visual control of reduction through a medial epicondyle osteotomy and to evaluate whether it is superior to the conventional approach in terms of clinical and radiological outcome. We hypothesize that besides the increased access morbidity of a medial epicondyle osteotomy optimized fracture reduction and thus better clinical outcome can be achieved.

## Materials and methods

The study was reviewed and approved by the local Institutional Review Board (IRB) (registered number: PV7319). All procedures were performed in accordance with the ethical standards of the Institutional Research Committee and with the Helsinki Declaration of 1964 and its subsequent amendments.

**Study patients:** All tibial plateau fractures treated between 2014 and 2021 from the in-house trauma registry were retrospectively reviewed and classified according to the AO/OTA classification and the 10-segment classification [12, 16]. Patients treated with medial epicondyle osteotomy (med ECO) and fulfilling the inclusion criteria, including the availability of pre- and postoperative CT imaging, were included in the study cohort. For comparison, 14 patients without medial femoral epicondyle osteotomy were selected from the institutional database based on comparable demographic characteristics and fracture patterns. A larger control group was initially considered; however, due to the rarity and heterogeneity of severe medial tibial plateau fractures, it was not possible to identify additional cases with sufficiently comparable injury patterns. Therefore, only the most comparable cases were included to allow for a clinically meaningful comparison. Comparability between groups was addressed by considering demographic variables (age, sex, BMI) and fracture characteristics, including soft tissue injury (Gustilo classification) and fracture pattern (AO/OTA and 10-segment classification). Exclusion criteria included patients aged < 18 years, comminuted bicondylar tibial plateau fractures treated with osteotomy of the lateral epicondyle, such as patients undergoing primary total knee arthroplasty treatment, and cases without both pre- and postoperative CT scans.

Additionally, patients treated with isolated lateral femoral epicondyle osteotomy without concomitant medial epicondyle osteotomy were excluded, as these cases did not address the study question focusing on medial tibial plateau exposure. In contrast, patients who underwent combined medial and lateral epicondyle osteotomies were included, provided that medial epicondyle osteotomy was performed to address the medial fracture components and constituted the primary focus of treatment.

**Surgical management and postoperative treatment:** In all cases, fractures were fixed with internal plating using a posteromedial approach [17]. Lateral tibial plateau fractures were treated with an anterolateral or extended lateral approach and supportive plating [18, 19]. When necessary, particularly in polytraumatized patients or those with multiple concomitant injuries, external fixation was performed prior to open reduction and osteosynthesis. CT scans of the lower leg were performed within the first 72 h after surgery to assess postoperative joint congruency. Guided exercise started 48 h after surgery with passive joint motion in the supine position. Additional peripheral nerve block anaesthesia was applied. Patients were instructed to limit weight bearing to 20 kg and range of motion (0-0-90°) for at least 6 weeks.

**Surgical technique:** The extended medial approach represents a modification of the posteromedial approach described by Lobenhoffer et al., allowing extended exposure depending on fracture morphology. The procedure was primarily performed with the patient in supine position with their knee slightly flexed; however, in selected cases with predominant posterior fracture components, a prone position was used to facilitate access. A 6–10 cm skin incision was made along the dorsomedial tibial border. The pes anserinus tendons, which cross the incision transversely and lie over the medial collateral ligament, were retracted distally and medially using a small hook. The deep capsuloligamentous layer, which was subsequently exposed, was composed of the medial collateral ligament and the trapezoid-shaped posterior oblique ligament attached to it. Depending on the fracture location, the posteromedial or anteromedial tibia was then exposed by making a longitudinal incision in the deep capsuloligamentous layer parallel to the palpable tibial margin. The incision was made at the point where the medial collateral ligament meets the posterior oblique ligament. It was terminated proximally just below the joint line to avoid injury to the medial meniscus [17].

**Fig. 1:**
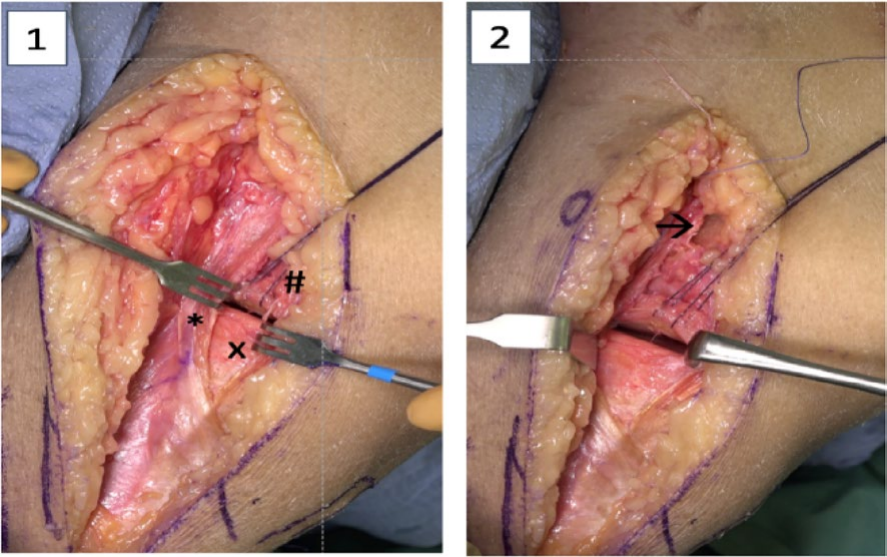
Surgical exposure using the extended medial approach with medial femoral epicondyle osteotomy. Intraoperative view of the extended medial approach with medial femoral epicondyle osteotomy in a bicondylar tibial plateau fracture (AO/OTA 41-C3.3). **1** Standard medial exposure before osteotomy, demonstrating the limited visualization of the medial articular surface. The superficial medial collateral ligament (MCL) has been retracted. **2** Exposure after medial femoral epicondyle osteotomy. The osteotomized insertion of the medial collateral ligament and posterior oblique ligament has been mobilized, creating an osteotomy window (arrow) that substantially improves visualization of the medial tibial plateau and facilitates reduction of complex fracture components. (X = tibia; # = femur; * = retracted superficial MCL; arrow = osteotomy window.)

The extended medial approach was performed with a medial epicondylar osteotomy which was performed as the wide MCL limited surgical exposure of the medial tibial plateau: a minimal 2 × 2 × 1 cm bone block containing the femoral insertions of the MCL and posterior oblique ligament was obtained [20, 21]. After proximal retraction of the medial meniscus, almost the entire medial tibial articular surface was exposed (Fig. 1). The decision to perform the medial epicondyle osteotomy was made intraoperatively based on fracture morphology and insufficient visualization using standard approaches, particularly in cases involving complex anteromedial and posteromedial fracture components.

Final fixation was achieved with subcortical screws using jail technique and either an isolated anteromedial or in combination with a posteromedial support plate. The medial femoral epicondyle osteotomy was reduced and fixed with two cortical or cancellous screws after articular reduction and fixation [19, 20, 22].

**Radiological analysis:** Multiplanar reconstructed postoperative CT scans were used to assess reduction quality by two fellowship trained trauma surgeons. Fracture step and gap were measured in the plane demonstrating the maximum displacement of the articular surface within the medial tibial plateau using standard PACS measurement tools. Radiological evaluation was performed independently and in cases of discrepancy, a consensus reading was obtained. Blinding to the surgical technique was not feasible due to the visibility of implants and the osteotomy on postoperative imaging.

In all segments of the medial plateau, fracture steps, comminution and fracture gaps were considered. Postoperatively the medial to lateral width of the tibial plateau was measured at its widest diameter. The posterolateral slope was measured on sagittal CT sections corresponding to the posterolateral articular surface. This parameter was included as a general measure of overall sagittal alignment in complex bicondylar fracture patterns, although the primary focus of the study was on reduction quality and deformity correction of the medial tibial plateau [23]. In addition, the radiological Rasmussen score was evaluated to objectify the reduction status of the tibial plateau [24]. 

**Clinical follow-up evaluation and complication analysis:** The follow-up assessment was performed at a minimum of 2 years after the index surgery. The KOOS and clinical Rasmussen scores and the IKDC subjective knee form score were used to measure subjective and functional outcomes [24–27]. Early complications (within 3 weeks postoperatively) were defined nerve damage and early peri-implant infection. The cut-off values used to assess the quality of fracture repositioning were a fracture step of more than 2 mm [6]. A threshold of 5 mm for fracture gap was used as a pragmatic cut-off to identify relevant residual incongruities in complex fracture patterns, although this parameter is less well established in the literature.

Late complications (> 3 weeks postoperatively) were defined as material removal due to patient reported problems, required total knee arthroplasty (TKA), arthrolysis, leg axis osteotomy and ligament reconstruction.

**Statistical analysis:** The data were summarized using descriptive statistics. In addition, a comparative analysis between the med ECO and control group was performed to compare patients treated with med ECO with patients non-ECO clinically and radiographically. A chi-squared test was used to assess the significance of association between categorical variables by comparing observed and expected frequencies. Analyses were performed using GraphPad Prism 8 (San Diego, CA, USA). Statistically significant was defined as a significance level of α = 0.05 or less.

## Results

The retrospective analysis identified 15 patients treated with a medial epicondyle osteotomy (med ECO) who fulfilled the inclusion criteria (9 males, 6 females; mean age 50.9 ± 11.6 years) at two Level 1 trauma centers. Clinical follow-up including PROMs was available for 14 of these patients due to one case of loss to follow-up.

For comparison, 14 patients without medial femoral epicondyle osteotomy were selected as a control group based on comparable demographic characteristics and fracture patterns. Clinical follow-up was available for 13 patients in the control group due to one case of loss to follow-up.

**Fig. 2:**
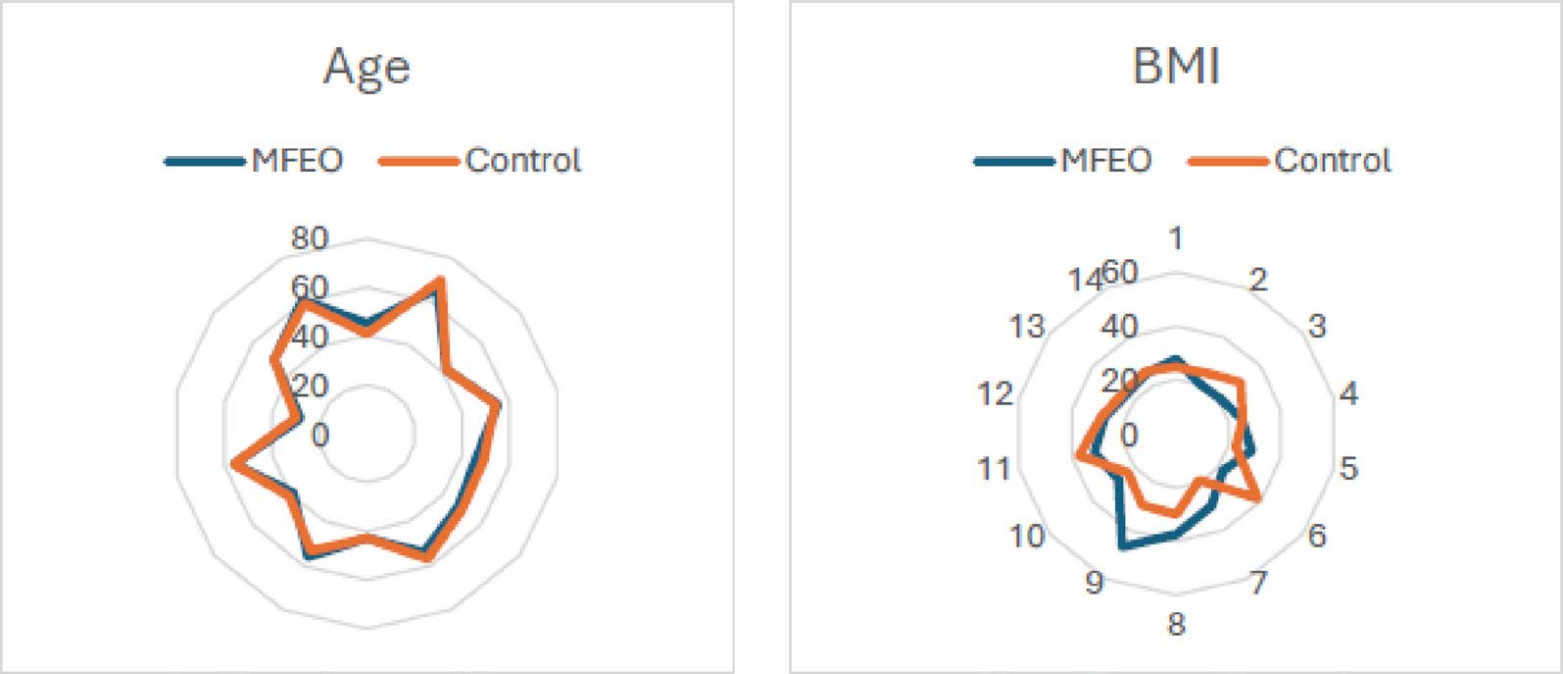
Baseline demographic characteristics. Regarding the demographic data, there was no significant difference between the two populations in the paired t-test statistical analysis

Figure 2 shows, that there were no significant differences in baseline demographic characteristics between the two groups (Fig. 2).

Overall, 15 patients (52%) were polytraumatized (according to Injury Severity Scale > 15) and 21 patients (72%) had to be treated with an external fixation (Table 1). One-third of the patients developed a compartment syndrome of the affected leg. All were closed soft tissue injuries and were treated after soft tissue consolidation. There was no difference in soft tissue state, or base line characteristics among both groups.

**Table 1:**
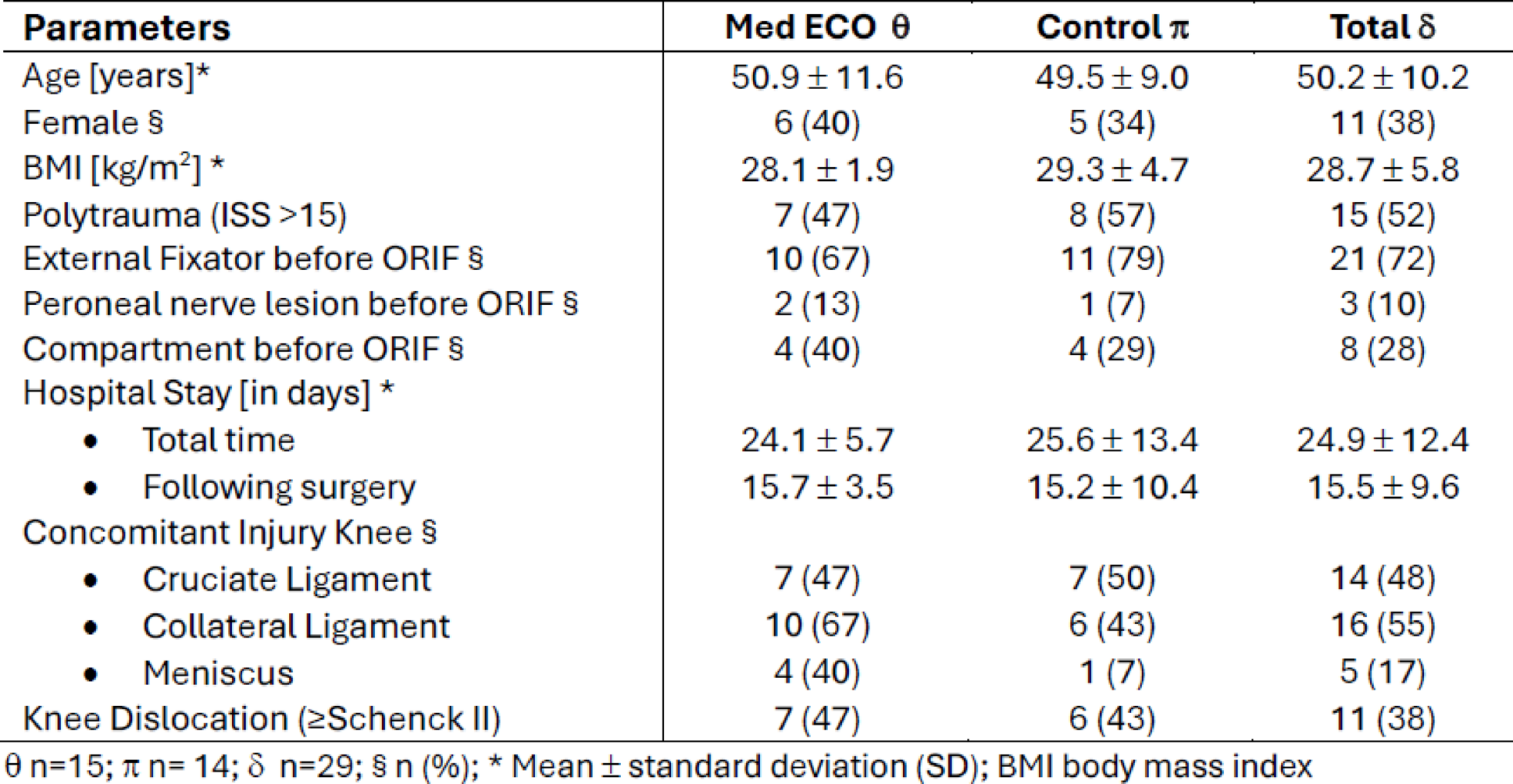
Demographics and preoperative characteristics

**Fig. 3:**
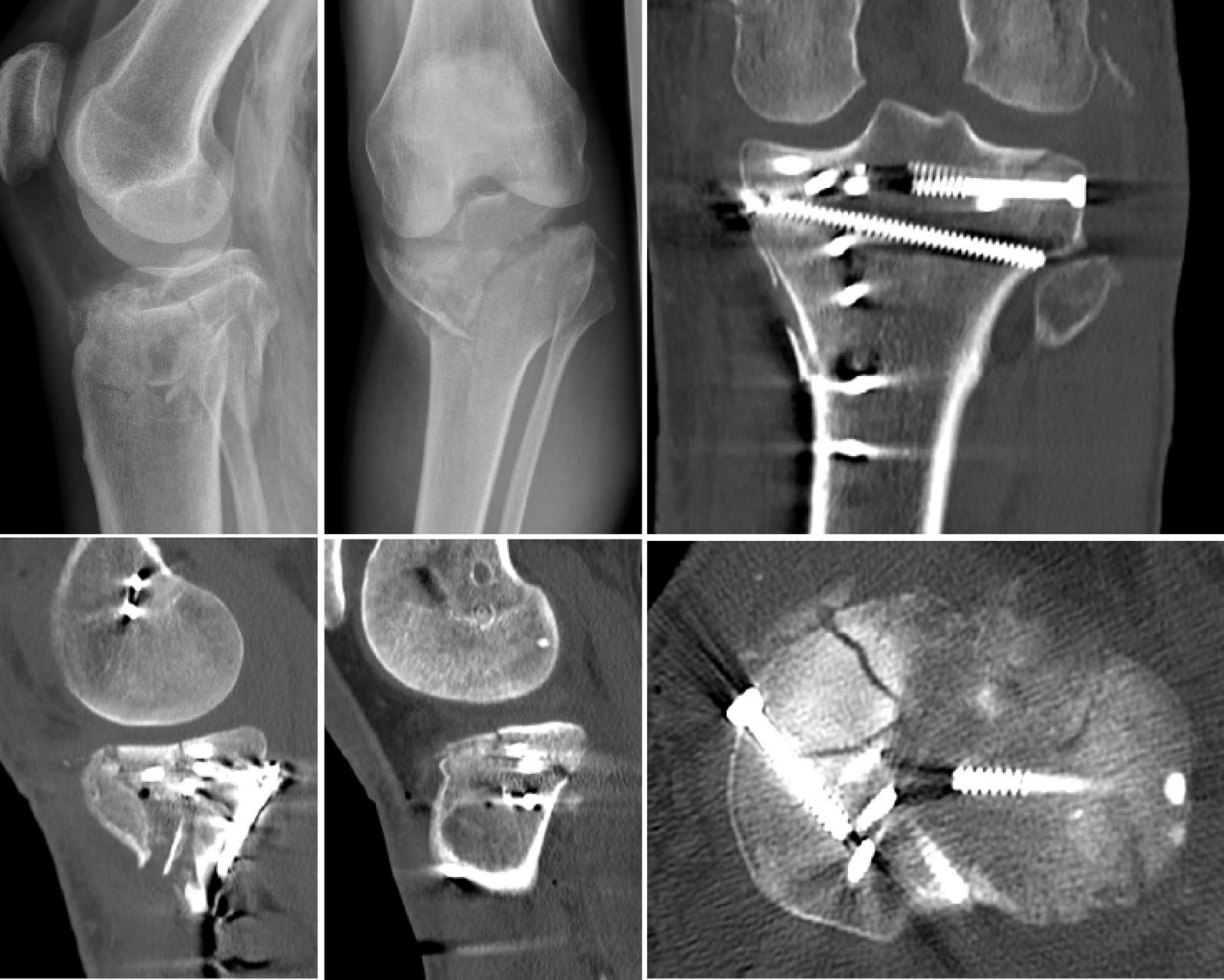
Pre- and postoperative imaging of a bicondylar tibial plateau fracture treated with osteotomy of the medial epicondylus. 42-year-old carpenter with hyperextension tibial plateau fracture with an acute compartment syndrome without neurovascular injury after a 150 kg pillar hit his upper thigh.** A–B**: Preoperative presentation of an AO/OTA 41-C3-3 bicondylar tibial plateau fracture (**A**: sagittal,** B**: coronar). All 10 segments are involved [12].** C–F**: Post-operative CT control after anterolateral and medial plate osteosynthesis

There was no significant difference between the number of AO/OTA type B and C fractures, although type B3 fractures were only present in the med ECO group. Type B3 fractures, particularly OTA type B3.3, which were split depression fractures of the medial plateau or involving the tibial spines of the medial plateau, accounted for 27% of cases treated with med ECO and were all associated with a concomitant knee dislocation. A review of the affected segments revealed that, on average, at least three of the four medial segments and three of the four lateral segments were involved (Table 2). All cases of pre-operative peroneal nerve injury resolved without the need for additional surgery. Figure 3 shows pre- and postoperative imaging of a bicondylar tibial plateau fracture treated with med ECO.

**Table 2:**
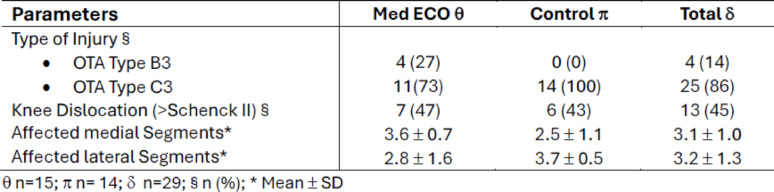
Preoperative characteristics

For procedures including med ECO, the mean duration of surgery was found to be shorter (323.5 ± 134.9 min) than for conventional surgery (367.5 ± 118.5 min; *p* > 0.05), with a total mean duration of 345.5 ± 126.7 min. The surgical procedures were conducted with the patient in a variety of positions. Most cases were conducted with the patient in a supine position (83%), with fewer cases conducted in a prone position (17%). A comprehensive overview of the surgical approach, patient positioning, and the technique used to expose the medial and lateral plateau is provided in Table 3.

**Table 3:**
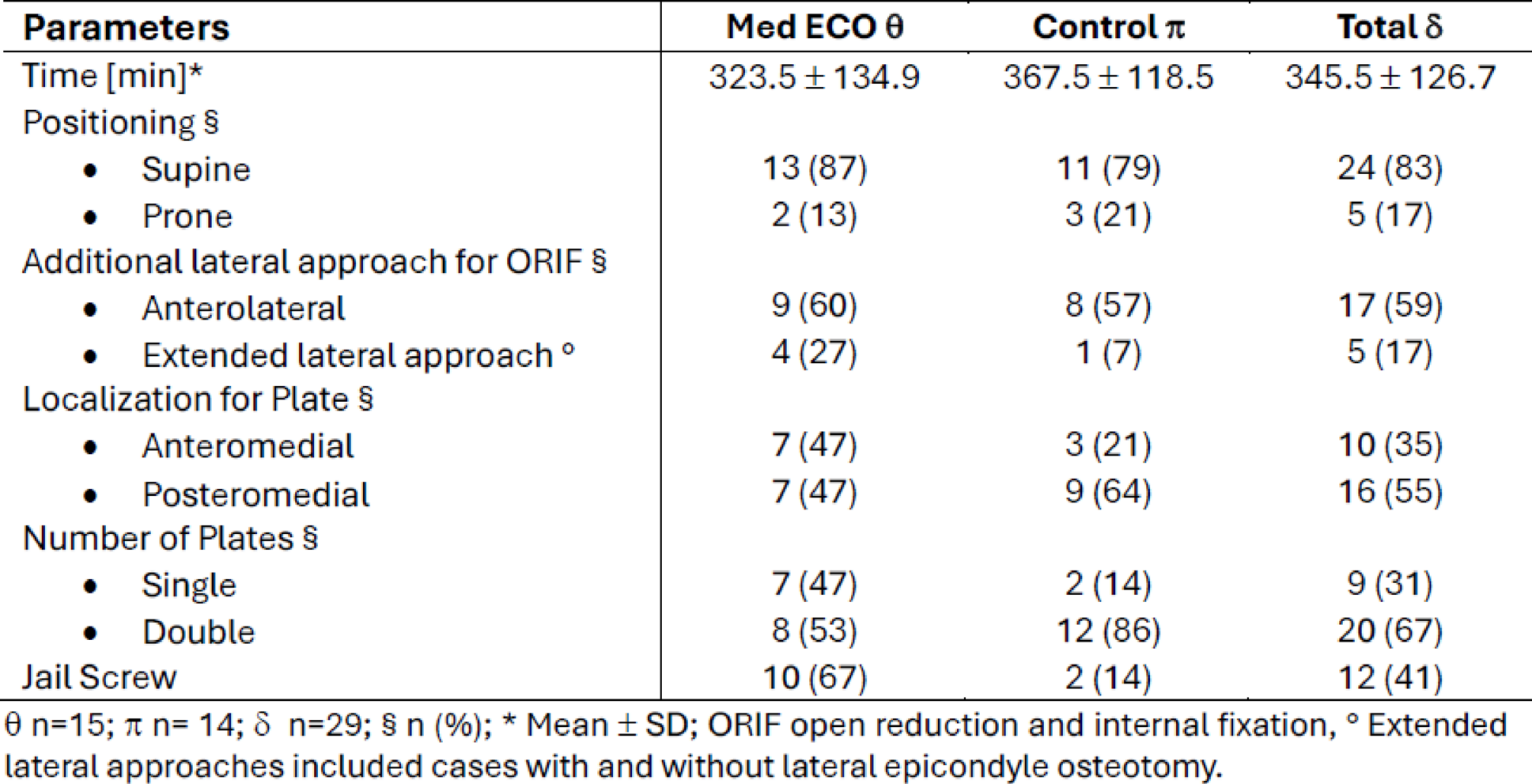
Operative characteristics

As detailed in Table 4, the radiological results indicate that there is no significant difference in postoperative depression in the medial articular surface between cases with osteotomy (33%) and without (34%), but with fewer gaps in the former (20 vs. 34%). Following surgery, the physiological slope did not differ significantly between the med ECO and control groups (*p* > 0.05). The radiological Rasmussen score did not demonstrate a statistically significant difference.

**Table 4:**
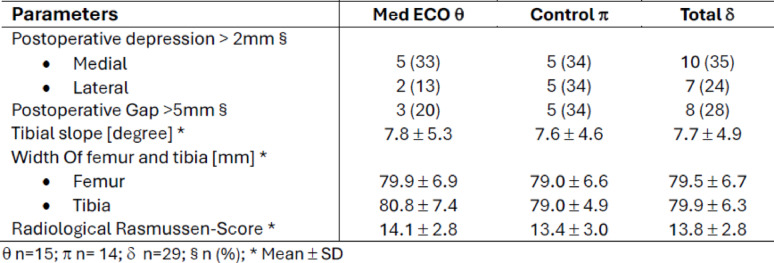
Radiological results

The results over a mean follow-up of 71.65 ± 18.9 months, together with the complications observed, are shown in Table 5. In terms of IKDC and KOOS scores, no significant statistical differences were found between both groups. No major osteotomy-specific complications were observed. In particular, there were no cases of delayed union or non-union, no fixation failures, no symptomatic hardware requiring removal related to the osteotomy, and no osteotomy-related reoperations. There was also no statistically significant association between the incidence of material removal due to patient-related complaints or the need for surgical revision due to malreduction in the form of knee arthroplasty, arthrolysis, leg axis osteotomy or ligament reconstruction between the groups. Overall, the postoperative ability to participate in sports and recreational activities was low in both groups represented as one subcategory in the KOOS score of 23.0 ± 19.0.

**Table 5:**
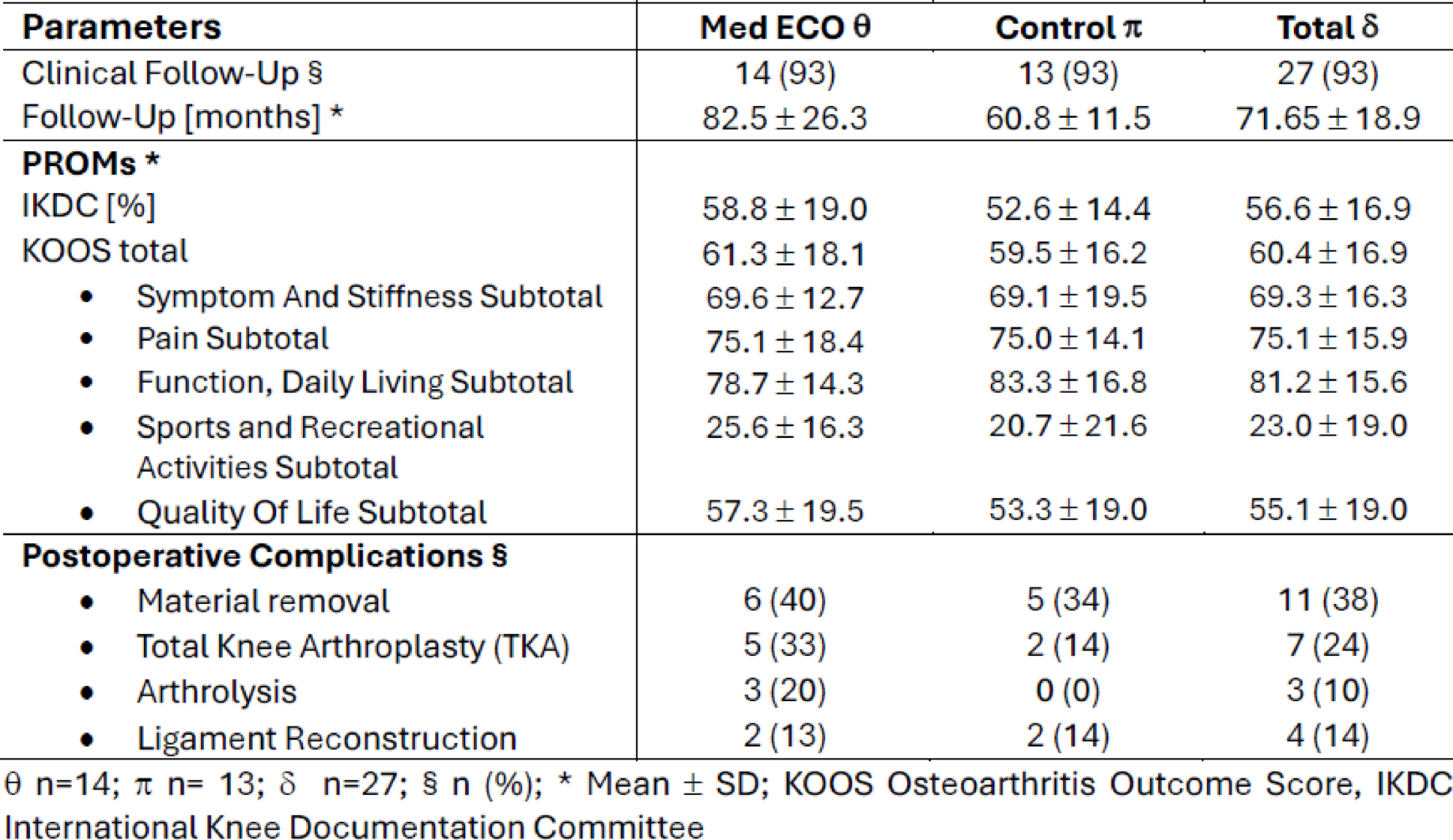
Clinical outcome

## Discussion

The main finding of our study is that good to fair clinical and radiological results were achieved overall using an extended medial approach with a medial femoral epicondyle osteotomy in highly complex tibial plateau fractures. The retrospective comparative cohort analysis revealed slight, though not significant, differences in radiographic or clinical outcomes between the cohorts. The results of this study must be interpreted with caution, as the retrospective design and the indication-dependent use of medial epicondyle osteotomy introduce a relevant risk of confounding by indication, thereby limiting comparability between groups. The higher number of involved medial segments in the med ECO group reflects increased fracture complexity, which likely influenced the indication for osteotomy and constitutes an additional source of confounding. This should be taken into account when interpreting the comparative findings. Differences in fixation strategy between groups likely reflect fracture-specific surgical decision-making and may have influenced radiological and clinical outcomes.

The cohort analyzed represents an exceptionally severe injury population, with more than 70% of all cases classified as AO/OTA type C3 fractures. These injuries were frequently accompanied by extensive soft-tissue trauma, compartment syndrome, neurovascular compromise, peripheral ligament injuries, or the need for initial external fixation. The association between fracture severity and clinical outcome has been highlighted in several studies [6, 15, 28–32]. Beisemann et al. demonstrated that achieving a reduction of less than 2 mm significantly improves clinical outcomes, underscoring the importance of precise anatomical reconstruction [28]. In line with this, other authors have suggested that the accuracy of fragment repositioning is as crucial for functional recovery as the fracture severity itself, which may explain why more severe fractures, being inherently more difficult to reduce, often result in poorer outcomes [6, 29]. In contrast to our findings, some studies have reported no clear correlation between injury severity and clinical results, indicating that the relationship between fracture complexity, reduction quality, and long-term function remains multifactorial [31, 32]. 

Two recent studies that investigated the effect of a lateral epicondyle osteotomy. Both studies demonstrated that the severity of tibial plateau fractures, as classified by the AO/OTA system, was strongly associated with functional outcome, even when an excellent anatomical reduction had been achieved [15, 30]. In the study by Behrendt et al., patients with more complex AO/OTA type C fractures showed significantly lower KOOS, Lysholm and IKDC scores compared with those with type B injuries, despite similarly good radiological alignment [15]. Fahlbusch et al. likewise reported that patients with highly complex fractures (types B3/C3) achieved better joint congruency using an extended approach with epicondylar osteotomy, yet their mean IKDC scores still reflected the negative impact of higher fracture complexity [30]. 

Existing literature indicates that approximately 8–10% of all tibial plateau fractures involve the medial articular surface, making complex medial patterns uncommon and usually limited to simple split fractures [25]. Owing to the typical morphology of tibial plateau injury patterns, the need for a medial epicondylar osteotomy arises predominantly in the context of more complex fracture configurations. In particular, fracture patterns involving the anteromedial and posteromedial segments, as well as multicolumnar fractures with a predominant medial deformity component, appear to benefit most from extended medial exposure. These configurations are often associated with limited visualization using standard approaches and may therefore represent the primary indication for medial epicondyle osteotomy in clinical practice [33, 34]. 

In this study > 70% of the fractures were classified as type C AO/OTA types. In accordance with other studies, these fracture patterns were frequently accompanied by severe soft-tissue injuries, and the outcome of a tibial plateau fracture depends not only on the quality of fracture reduction but also on the extent of associated ligamentous, cartilaginous, and meniscal damage, as suboptimal treatment can lead to poor knee function and osteoarthritis. A systematic review of 18 studies involving 877 patients reported soft-tissue lesions in 93% of cases, most commonly affecting the lateral meniscus, anterior cruciate ligament and lateral collateral ligament [35]. These circumstances significantly compromised the clinical outcomes of patients treated with a medial epicondylar osteotomy, making it difficult to clearly discern the potential benefit of the procedure. Furthermore, the authors note that type C tibial plateau fractures are highly variable between individuals, making a comparative difficult both to implement and to interpret.

Nevertheless, the present study provides a comprehensive analysis of the clinical outcomes and practical applications of a medial epicondyle osteotomy in complex tibial plateau fractures. The findings of this study demonstrate that the procedure appears to be safe and allows adequate exposure and reduction and does not result in any cases of medial instability. Several studies that specifically evaluated outcomes of particularly high-grade bicondylar and C3-type injuries, consistently highlight the decisive role of adequate visualization and precise reduction. Rosteius et al., who analyzed OTA type B and especially C fractures, found that even minimal residual steps significantly impair functional outcomes, identifying threshold values of roughly 2–3 mm beyond which IKDC and Lysholm scores decline markedly [6]. In a cohort dominated by severe bicondylar patterns, Kraml et al. similarly reported long-lasting functional deficits with consistently lower KOOS subscores on the injured side and a 10-year arthroplasty rate exceeding 12% [34]. Likewise, Raschke et al. demonstrated that in complex C3-type lateral plateau fractures, the fracture’s location relative to the posterolateral complex determines whether standard approaches provide sufficient visualization; posteriorly extending fracture lines frequently required extended approaches to achieve anatomical reconstruction [33]. Collectively, these studies underscore that outcomes in complex tibial plateau fractures depend heavily on the surgeon’s ability to obtain adequate exposure and restore articular congruity with high precision. Our results demonstrate that good to fair radiological and clinical outcomes can be achieved using the extended medial approach, facilitated by osteotomy of the femoral insertion of the MCL and POL and mobilization of the medial meniscus, substantially improves visualization of the anteromedial and posterior-medial tibial plateau in these extremely complex situations, too: More than 80% of patients had good to excellent postoperative radiological results, as defined by the radiological Rasmussen score, indicating a significant improvement in radiological parameters. Cadaveric studies have already shown that visualization can increase from approximately one-third of the articular surface with standard anteromedial approaches to over 60% with med ECO [12, 27, 34]. Our clinical observations are consistent with these experimental findings: improved visualization appears to contribute meaningfully to reduction quality, with patients treated using med ECO tending to have fewer residual gaps > 5 mm. While medial epicondyle osteotomy improves visualization of the articular surface, adequate control of fracture deformity depends primarily on the fixation strategy [17, 19]. In complex fracture patterns, particularly those involving anteromedial and posteromedial components, insufficient fixation may limit deformity control despite improved exposure [17, 19, 33]. 

Despite the good radiographic results, the clinical outcomes, quantified by the IKDC- score and the KOOS, were only good to fair at follow-up. It is important to note that these scores assess not only knee function and mobility but also depend on the patient’s overall health. Because nearly 50% of the patients sustained their tibial plateau fracture as part of a polytrauma, their additional injuries may have adversely influenced their clinical scores. When comparing our results with other outcome studies focusing on complex bicondylar and C3-type fracture patterns, our mean total KOOS lies slightly below the mid-term KOOS of 67.84 reported by Jansen et al. and also below the KOOS presented by Kraml et al., whose subscores for *Activities of Daily Living* and *Sports* values reached 84.3 and 59.5, respectively [36, 37]. In contrast, our KOOS subscores reflect the markedly higher injury severity in our cohort, in which massive soft-tissue trauma and polytrauma were common and are known to substantially limit functional recovery. Our mean IKDC score also aligns with the findings of Rosteius et al., who reported a mean IKDC of 62.7 in OTA B/C fractures and demonstrated that even small residual steps or gaps significantly reduce functional outcomes [6]. Although Raschke et al. did not report functional outcome scores, their observation that posteriorly extending fracture patterns often require extended approaches underscores the technical difficulty of these injuries and helps to contextualize the moderate functional results consistently reported across all studies, including ours [33]. These findings suggest that not only the fracture morphology and the need for extended exposure, but particularly the severity of accompanying soft-tissue injuries, exert a decisive influence on the achievable functional outcome in this patient population.

Importantly, the present findings primarily support the feasibility and safety of medial epicondyle osteotomy rather than demonstrating a clear clinical or radiological superiority over conventional approaches. Taken together, our data suggest that med ECO enables precise reduction of the medial tibial plateau when standard visualization is insufficient. Given the sevenfold increased risk of post-traumatic osteoarthritis described by Snoeker et al., accurate anatomical reconstruction remains crucial for long-term outcomes [38]. However, the clinical benefit of improved visualization may not fully translate into mid-term functional scores when fracture severity and soft-tissue compromise dominate the clinical course. This may reflect that, in highly complex fracture patterns, improved exposure alone is insufficient to overcome the impact of fracture severity and associated soft-tissue injury on clinical outcomes.

## Limitation

This study is subject to several limitations. Primarily, the sample size was modest and comprised a variety of complex fracture patterns. Although the study employed rigorous inclusion and exclusion criteria, heterogeneity persisted among the study group due to differences in injury characteristics and surgical techniques, which limits the comparability between groups. Nearly all patients suffered multiple associated injuries, frequently severe, such as pelvic trauma, contralateral lower-extremity fractures, ligament ruptures, or polytrauma. These injuries influenced treatment decisions, timing of surgery, postoperative mobilization, and rehabilitation protocols, thereby limiting the interpretability of clinical outcomes.

In addition, the retrospective design and the indication-dependent use of medial epicondyle osteotomy introduce a relevant risk of confounding by indication. The decision to perform med ECO was primarily based on fracture complexity and the need for extended visualization, which further limits the comparability between groups and should be considered when interpreting the results.

Furthermore, although a comparative cohort design was used to control for confounders, the variability inherent in C3 fractures restricts the accuracy of any matching methodology. Even fractures categorized identically according to AO classification may differ substantially in fragment size, depression depth, comminution, and soft-tissue involvement. Thus, the comparative analyses should be interpreted as approximations rather than precise representations of the isolated osteotomy effect. Moreover, formal interobserver reliability was not assessed, and blinding of assessors was not feasible due to the nature of the imaging, which may introduce measurement bias.

Long-term outcomes, including post-traumatic osteoarthritis and conversion to total knee arthroplasty, were not assessed and require further investigation with longer follow-up periods. Given that tibial plateau fractures, especially C3 patterns, are known to carry a substantially increased risk for osteoarthritis, future research should include long-term radiological and clinical monitoring to better determine the impact of med ECO on joint preservation.

## Conclusion

Medial epicondylar osteotomy is a feasible and safe technique that allows improved visualization and facilitates anatomical reduction in complex medial tibial plateau fractures. However, no clear clinical or radiological advantage over conventional approaches was observed. Therefore, its use should be considered selectively in fracture patterns requiring extended medial exposure, taking into account the overall fracture complexity and associated soft-tissue injury.

## Data Availability

The datasets generated and/or analysed during the current study are available from the corresponding author on reasonable request.

## Abbreviations

BMI: Body mass index
CT: Computed tomography
eM: Extended medial approach
IRB: Institutional Review Board
IKDC: International Knee Documentation Committee
ISS: Injury severity score
KOOS: Knee injury and osteoarthritis score
MCL: Medial collateral ligament
med ECO: Medial femoral epicondyle osteotomy
non-ECO: Conventional approach without medial epicondyle osteotomy
OA/OTA: Arbeitsgemeinschaft für Osteosynthesefragen/Orthopaedic Trauma Association
ORIF: Open reduction and internal fixation
PCL: Posterior cruciate ligament
POL: Posterior oblique ligament
PROMs: Patient reported outcome measures
SD: Standard deviation
TKA: Total knee arthroplasty
